# IL4/IL4R signaling promotes the osteolysis in metastatic bone of CRC through regulating the proliferation of osteoclast precursors

**DOI:** 10.1186/s10020-021-00411-2

**Published:** 2021-12-04

**Authors:** Qian Jin, He Yang, Zhao Jing, Wu Hong-hua, Song Ben-jing, Wang Li-ting, Ye Li-juan, Xu Wei, Kang Xia, Wu Juan, Zheng Wei

**Affiliations:** 1 Department of Orthopedics, General Hospital of Western Theater Command, Rongdu Avenue No. 270, Chengdu, 610000 People’s Republic of China; 2 College of Medicine, Southwest Jiaotong University, North Section 1 No. 111, Second Ring Road, Chengdu, 610000 People’s Republic of China; 3 Department of Pharmacy, General Hospital of Western Theater Command, Rongdu Avenue No. 270, Chengdu, 610000 People’s Republic of China; 4 Biomedical Analysis Center, Army Medical University, Chongqing, 400038 People’s Republic of China

**Keywords:** Bone metastasis, CRC, Osteoclast precursors, IL-4/IL-4R

## Abstract

**Background:**

Bone metastasis of colorectal cancer (CRC) often indicates a poor prognosis. Osteolysis can be observed in metastatic sites, implying an aberrant activation of osteoclasts. However, how osteoclastogenesis is regulated in metastatic microenvironment caused by colorectal cancer is still unclear.

**Methods:**

In this study, mice bone metastatic model of CRC was established through injection of MC-38 or CT-26 cells. BrdU assays showed primary CD115 ( +) osteoclast precursors (OCPs) proliferated at the first 2 weeks. Transcriptomic profiling was performed to identify differentially expressing genes and pathways in OCPs indirectly co-cultured with CRC cells

**Results:**

The expression of IL4Rα was found to be significantly upregulated in OCPs stimulated by tumor conditioned medium (CM). Further investigation indicated that IL-4 signaling regulated proliferation of OPCs through interacting with type I IL4 receptor, and neutrophils were the main source of IL-4 in bone marrow. The proliferation of OCPs can be inhibited in IL4 deficiency mice. In addition, ERK pathway was activated by IL4/IL4R signaling. Ravoxertinib, an ERK antagonists, could significantly prevent bone destruction through inhibiting the proliferation of OCPs.

**Conclusion:**

Our study indicates the essential role of IL4/IL4R signaling for the proliferation of OCPs in early metastasis of CRC predominantly through activating ERK pathway, which remarkedly impacts the number of osteoclasts in later stage and leads to osteolytic lesions. Moreover, Ravoxertinib could be a new therapeutical target for bone metastasis of CRC.

**Supplementary Information:**

The online version contains supplementary material available at 10.1186/s10020-021-00411-2.

## Background

Although the incidence of bone metastasis takes up only about 4–11% in total patients with colorectal cancer (CRC), it shows a quite powerless prognosis (Suresh Babu et al. 2017). The median survival of CRC patients with bone metastasis is only up to 6 months and the 1-year survival rate is only 30% (Lei et al. 2019; Kawamura et al. 2018). Unfortunately, due to low incidence, rare studies have focused on the regulation of bone metastasis from CRC and the mechanism beneath clinical features is poorly understood until now.

Osteolysis is a typical change during bone metastasis of CRC. It was reported that hypercalcemia and pathologic fractures, as the consequences of excessive bone resorption, were two independent factors of poor prognosis in patients with bone metastasis from CRC (Kawamura et al. 2018), indicating osteolysis was directly associated with the progression of CRC. It has been well known that the abnormal activation of osteoclasts is responsible for the formation of osteolytic lesions in bone metastasis of CRC (Clohisy and Ramnaraine 1998; D'Amico and Roato 2012; Hirashima et al. 2001; McCoy et al. 2013). Osteoclasts derive from monocyte/macrophage cell lineage. Monocyte/macrophage can commit to osteoclast result after differentiating into early osteoclast precursors (OCPs) (Teitelbaum and Ross 2003). The regulation of tumor microenvironment on OCPs or OCs has been deeply investigated in several tumors with high incidence of bone metastasis, such as breast cancer, lung cancer and prostate cancer (Aukes et al. 2017; Grano et al. 2000). Although our previous studies demonstrated that CRC cells could regulate osteoclastogenesis after bone metastasis of CRC (Zi-Chen et al. 2020; Qian et al. 2021), the process of how osteoclastogenesis is regulated by microenvironment of CRC is still far from clear.

Type 2 innate signals, such as IL-4 signal, are closely associated with tumor progression, macrophage polarization and osteoclastogenesis. It was reported that IL-4 could inhibit the osteoclast differentiation of monocytes. In addition, IL-4 could inhibit the expression of RANK in a STAT6-dependent manner (Moreno et al. 2003). Furthermore, it can also inhibit NF-κB-induced osteoclastogenesis through activation of PPARγ (Bendixen et al. 2001). After stimulating with IL-4, RAW 264.6 cells, a monocyte cell line, can present with features of macrophages than osteoclasts (Hu et al. 2014). These studies indicated that IL-4 negatively regulated the osteoclast differentiation. However, in some circumstances, such as deficiency of estrogen, IL-4 induced M2 macrophages were more likely to commit to osteoclastogenic result, comparing to lipopolysaccharide (LPS)/interferon γ (IFN-γ) induced M1 macrophages (Dou et al. 2018). During a period of tumor metastasis, IL-4 could induce the production of MMP-9, an osteolytic cytokine, and the M2 polarization within tumorigenic microenvironment to promote metastasis of breast cancer (Khabbazi et al. 2015). These results implied that IL-4 may promote the osteolysis in special microenvironments through some mechanisms other than directly regulation of osteoclastogenesis. Until now, the role of IL-4 signals in bone metastasis, especially in bone metastasis of colorectal cancer, is barely comprehended.

In this study, we firstly found systemic knockout IL-4 can prevent the osteoclast formation and bone destruction in bone metastasis of CRC. Then, we identified that the effect of IL-4 on inhibiting osteoclast formation depended on stimulating the proliferation of CD115-positive osteoclast precursors but not osteoclast differentiation. Mechanically, IL4/IL4Rα signaling was indispensable to regulate the proliferation of osteoclast precursors in bone metastasis from CRC through activation of Erk pathway both in vitro and in vivo. Interestingly, we found administration of Ravoxertinib, an inhibitor of ERK pathway, can significantly alleviate the IL4/IL4Rα-induced activation of osteoclasts and bone resorption. These results reveal a novel mechanism that IL4/IL4Ra signaling impacts the proliferative stage of preosteoclasts and therefore Ravoxertinib could be a potential therapeutic in the treatment of patients with bone metastasis from CRC.

## Materials and methods

### Animal experiments

All procedures using experimental animals were approved by the Institutional Animal Care and Use Committee at General hospital of Western Theater Command. C57BL/6 or BALB/c male mice at 6 to 8 weeks old were used for experiments. 10 mice were maintained in a ventilated cage. Mice were kept under a 12-h light/dark cycle. IL-4 knockout (IL-4^KO^) mice were purchased from Shanghai Model Organisms Center Inc. and were maintained in our animal facility.

To establish the bone metastasis model of CRC, 500,000 of MC-38 cells or CT-26 cells were injected into tibia following standard procedures (Campbell et al. 2012). To inhibit the expression of IL4Rα in vivo, 10 μg IL4R siRNA (siIL4Rα) or negative control (scRNA) were mixed with INVI DNA RNA Transfection reagent (Invigentech, CA, USA) for 15 min at room temperature (RT) according to the manufacturer’s instructions, and then was injected intratibially once a week. For blockage of ERK pathway in vivo, Ravoxertinib (10 mg kg^−1^) was injected into tibias once a week. For blockage of IL-4 in vivo, 0.25 mg IL-4 neutralized antibody (Biolegend, USA) was injected into tibias twice per week. The number of mice used for in vivo experiments was indicated in each figure.

### Early OCPs isolation and cell culture

Bone marrow was rinsed out using sterilized PBS and centrifuged. Then the cell precipitation was resuspension and filtered through 100 μm filter. After centrifuged again, precipitation was resuspended and labelled with CD115-APC antibody (Biolegend) and RANK-PE antibody (Biolegend) for 30 min after erythrocyte lysis. Freshly isolated CD115( +) early OCPs through FACS by using FACSAria III (BD Biosciences) were then cultured in DMEM containing 10% FBS and 2% Penicillin–streptomycin solution with 50 ng ml^–1^ colony stimulating factor (M-CSF, Abcam). After culture for 2 days, the culture medium was changed, and the cells were used for subsequent experiments. For IL-4 stimulation, early OCPs were treated with IL-4 (50 ng/ml) as well as M-CSF (50 ng ml^–1^) for 48 h. For collecting conditioned medium (CM), MC-38 cells were cultured and when the confluent ratio was about 85–90%, the culture medium was replaced with DMEM, and the cells were continued to culture for 24 h, then the culture medium was collected and stored at − 20℃. When stimulating early OCPs by CM, 3.0 × 10^5^ of early OCPs was seeded in 12-well plates and the CM was added into culture medium at a ratio of 1:1, DMEM was used as control. For transfection with siRNA or treatment with inhibitors, 2.0 × 10^5^ of early OCPs was seeded in 12-well plates and cultured for 48 h. siRNA or controls were mixed with INVI DNA RNA Transfection reagent (Invigentech, CA, USA) for 15 min at RT, and then added into culture medium for 24 h. For treatment with antagonists, Ravoxertinib (110 nM), LY294002 (2 mM) or Oclacitinib (15 μM) were added into culture medium for 8 h. The IL4Rα siRNA sequence: RE: UUCUUCCAGAUGAUCAGCCTT; FW: GGCUGAUCAUCUGGAAGAATT.

### Proliferation assays

For in vitro experiments, Phase-Flow BrdU kit (Biolegend) was used, and the procedures were performed following manufacturer’s instruction. Briefly, BrdU solution was added into culture medium at a final concentration of 10 μg/ml and cultured for 2 h before collecting samples. Then the cells were collected, fixed, permeated and treated by DNase for 1 h at 37 ℃. BrdU antibody (1:100) was added and treated for 30 min at 4 ℃ in dark. For in vivo experiments, BrdU (100 mg kg^−1^) was injected intraperitoneally at 12 h and 3 h before collecting samples, respectively. Then the cells in bone marrow was collected following the same procedures as used in FACS. After surface markers were stained, the cells were fixed, permeated and treated by DNase for 1 h at 37 ℃. Then the samples were stained with BrdU antibody (1:100) for 30 min in dark.

### Apoptosis assays

When the apoptosis cells were detected by using TUNEL assays, In Situ Apoptosis Detection Kit (Roche) was used and the procedures were following the manufacturer’s instructions. Briefly, cell suspension was stained for extracellular markers of OCPs. Then, samples were incubated in TUNEL mixture for 1 h at 37℃ after fixed and permeated. After washing with PBS to remove excessive staining solution, the cell suspension was analyzed by flow cytometry as described above.

### RNA-Seq analysis

Early OCPs were treated with MC-38 CM for 48 h in the presence of M-CSF (50 ng ml^−1^) for 48 h. The samples were collected, and RNA was extracted by using TriZol. TruSeq™ RNA sample preparation Kit (Illumina) was used for preparing RNA-Seq transcriptome library and sequenced with IlluminaHiSeq xten (2 × 150 bp read length). The profiling data were analyzed on the free online platform of Majorbio Cloud Platform (www.majorbio.com).

### Flowcytometry analysis

The cell suspensions from bone marrow were collected as described above. The cells were incubated in desired antibodies for 30 min in 4 ℃. The antibodies used was anti-mouse CD115 conjugated with APC (Biolegend), anti-mouse IL4Rα conjugated with PE (Biolegend), anti-mouse F4/80 conjugated with APC (Biolegend), anti-mouse Ly6G conjugated with APC-Cy7 (Biolegend), anti-mouse Siglec F conjugated with PE (BD Biosciences),and anti-mouse IL-4-AF488 (Biolegend). The stained samples were detected by flow cytometry (BD FACSCalibur, BD Biosciences). The data was analyzed by using FlowJo v10 (Flowjo, LLC.).

### Histochemistry, immunofluorescence and imaging

Tibias were fixed in 4% paraformaldehyde (PFA) for 4 days, then the samples were washed and decalcified in a solution of 10% EDTA for 2 weeks and embedded in paraffin. For histochemistry, decalcified tibial cytosections were stained with tartrate resistant acid phosphatase (TRAP) staining (Wako) following manufacturer’s procedures. Briefly, 0.5 mL of TRAP stain solution was added on each section and incubated for 30 min at RT. After washing sections, adding 0.1 M AMPD-HCl buffer (pH 9.4) to soak the sections for 10 min. Then, removing excess moisture on the slides. For Safranin O-Fast Green staining, sections were immersed in 0.1% Safranin O solution for 3 min following by being immersed in 0.1% Fast Green solution for 10 s, and 1% acetum was utilized for color separation. After wash, dehydration and transparency, the sections were sealed with neutral resins. The images were captured by a fluorescence microscope IX81 (Olympus, Japan).

### RT-PCR analysis

Total RNA was isolated and performed using TRIzol reagent. Then RNA was reversely transcribed into cDNA by using RevertAid First Strand cDNA Synthesis kit (Thermo Fisher Scientific) following the manufacturer’s procedures. The mRNA levels were normalized to GAPDH. Relative target gene expression was calculated using the 2-ΔΔCq method. The primer sequences used for PCR were listed as below: GAPDH (RE: TGTAGACCATGTAGTTGAGGTCA; FW: AGGTCGGTGTGAACGGATTTG), Il4 (RE: and FW:), Il4Rα (RE: GAACAGGCAAAACAACGGGA; FW: ACGTGGTACAACCACTTCCA), Il13Rα (RE: GCGGACTCAGGATCACCTTC; FW: AGGTGGGCTCTCAGTGTAGT).

### Western blots

Proteins (50 µg) were separated using SDS-PAGE gels. Then the proteins were transferred to polyvinylidene difluoride (PVDF) membranes (Bio-Rad Laboratories). The PVDF membranes were then blocked with 5% BSA diluted in TBS for 1 h at room temperature. Primary antibodies were incubated according to the manufacturers' protocols. The samples were agitated at 4 ℃ overnight. Secondary antibodies were added and incubated at room temperature for 2 h. The densitometric analysis was performed using ChemiDoc Touch Imaging System (Bio-Rad Laboratories). The primary antibodies used are as below: rabbit anti-mouse GAPDH antibody (Cell Signaling Technology), rabbit anti-mouse β-actin antibody (Affinity), rabbit anti-mouse ERK antibody (Affinity), rabbit anti-mouse STAT6 antibody (Affinity), rabbit anti-mouse AKT antibody (Affinity), rabbit anti-mouse phosphorylated-ERK antibody (Affinity), rabbit anti-mouse phosphorylated-STAT6 antibody (Affinity), rabbit anti-mouse phosphorylated-AKT antibody (Affinity).

### ELISA assays

The detection of protein level of IL-4 was analyzed by ELISA assay using IL-4 ELISA detection kit (Biolegend) following manufacturer’s protocol. Briefly, the samples were placed at room temperature for 30 min. 50 μl Assay buffer A was added into each well following by adding 50 μl diluted samples and standards, and incubated at room temperature for 2 h. Then after the solution was discarded, 100 μl detection antibody solution was added per well and incubated at room temperature for 1 h following by adding 100 μl avidin-HRP A Solution. Then, 100 μl Substrate Solution F was added and incubated for 15 min in dark following by adding 100 μl Stop solution. The absorbancy was detected at 450 nm.

### μCT analysis

For μCT analysis, Skyscan1174 X-Ray Microtomograph (Bruker, Belgium) with an isotropic voxel size of 12.0 μm was used to image the whole tibias. Scans were conducted in 4% paraformaldehyde and used an X-ray tube potential of 50 kV, an X-ray intensity of 800 μA. For trabecular bone analysis of the proximal tibias, a region beginning at 0.1 mm below growth plate to the most distal end of the tibia was contoured. 3D images were obtained from contoured 2D images by methods based on distance transformation of the grey scale original images (N-Recon). 3D and 2D analysis were performed using software CT Analyser. All images presented are representative of the respective groups.

### Statistical analysis

Results are showed as means ± SD as required. Student's *t*-test was used in comparison of two groups. For more than two groups, one way analysis of variance (ANOVA) was used. Statistical significance was considered at P < 0.05. Ex vivo experiments were repeated at least 3 times.

## Results

### IL-4 deficiency attenuates bone resorption in bone metastasis of CRC

**Fig. 1:**
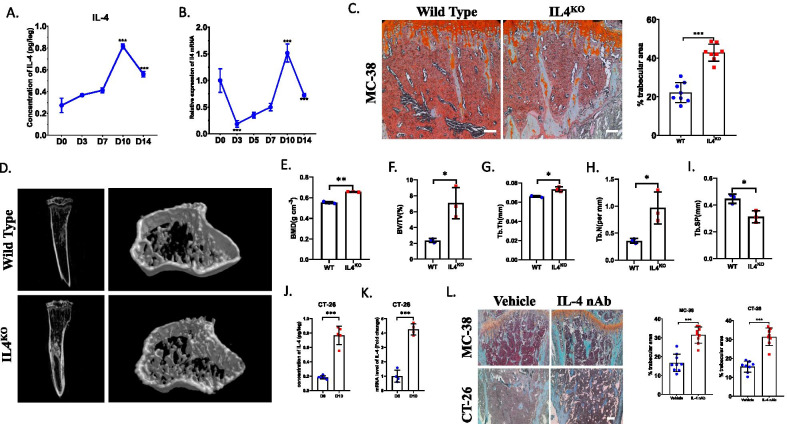
IL-4 deficiency attenuates bone resorption in bone metastasis of CRC. **A** ELISA assay detected the protein level of IL-4 in bone marrow at each timepoint after intratibially injection of MC-38 cells (n = 4, each sample was pooled from 2 mice). **B** qRT-PCR analysis showed the mRNA level of IL-4 at each timepoint (n = 3–5, each sample was pooled from 2 mice). **C** Histochemistry analysis showed the bone resorption in tibias at 21 DPI in wild type mice and IL4^KO^ mice (Scale bar = 100 μm) and quantification of trabecular area (n = 8). **D** μCT analysis compared the bone volume of tibias between wild type mice and IL4^KO^ mice at 21 DPI (n = 3). **E** Quantification of bone mineral density (BMD). **F** Quantification of trabecular bone volume fraction (BV/TV). **G**–**I** Quantification of trabecular thickness (Tb.Th) (**G**), trabecular number (Tb. N) (**H**), trabecular separation (Tb. Sp) (**I**) (n = 3). **J** ELISA assay detected the protein level of IL-4 in bone marrow after intratibially injection of CT-26 cells at D0 and D10 (n = 5, each sample was pooled from 2 mice). **K** mRNA level of IL-4 in bone marrow derived from tibias after injection of CT-26 at D0 and D10 (n = 4, each sample was pooled from 2 mice). **L** Histochemistry analysis showed the bone resorption in tibias at 21 DPI after injection of MC-38 cells or CT-26 cells in vehicle treated or IL-4 nAb treated groups (Scale bar = 100 μm) and quantification of trabecular area (n = 9). *p < 0.05, **p < 0.01, ***p < 0.001

To investigate whether bone resorption can be prevented in IL-4 deficiency condition, we established bone metastasis model of CRC through injection of MC-38 cells into tibias of wild type mice and IL-4 knocked out (hereafter named IL4^KO^) mice, respectively. Since we have demonstrated that obvious osteolysis began from 1 week post injection (Zi-Chen et al. 2020), we detected the mRNA and protein expression of IL-4 in bone marrow after injection of MC-38 until 2 weeks. The results showed the concentration of IL-4 increased gradually until 10 days post injection and then decreased (Fig. 1A). Consistently, the mRNA level of IL-4 also increased from D0 to D10 after injection of MC-38 cells (Fig. 1B). These results implied that IL-4 may potentially participate in the bone metastasis of CRC at early stage.

Then, we detected how depletion of IL-4 impacted the bone metastasis of CRC using IL4^KO^ mice. At 3 weeks post injection, the percentage of trabecular area in IL4^KO^ mice was restored compared to that of wild type (WT) mice (Fig. 1C). Consistently, μCT analysis revealed that cancellous bone was less destructed in IL4^KO^ mice than that in WT mice (Fig. 1D). Bone mineral density (BMD), BV/TV, Trabecular Thickness (Tb.Th) and Trabecular Number (Tb.N) were all higher in IL4^KO^ mice than those in WT mice (Fig. 1E–H). Contrarily, Trabecular separation (Tb.SP) was significantly lower in IL4^KO^ mice (Fig. 1I). These data indicated that systemic depletion of IL-4 could prevent bone loss in bone metastasis of CRC.

Next, we established bone metastatic model of CRC using a distinct CRC cell line, CT-26. ELISA analysis and qRT-PCR analysis showed the protein level and mRNA level of IL-4 also increased sharply at 10 days post injection compared to that in normal mice (Fig. 1J, K), indicating IL-4 may also participate in the bone metastasis of CT-26 cells. Then, we detected whether IL-4 could be a potential therapeutical target for treating osteolysis in bone metastasis of CRC. After injection of IL-4 neutralizing antibody intratibially, the bone osteolysis caused by MC-38 cells and CT-26 cells was significantly prevented (Fig. 1L), indicating blockage of endogenous IL-4 could attenuate bone resorption caused by CRC.

### Neutrophils-derived IL-4 stimulates the proliferation of early OCPs

**Fig. 2:**
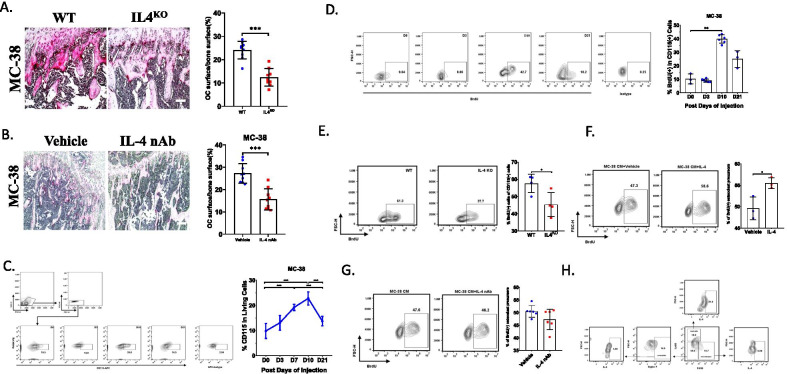
Neutrophils-derived IL-4 stimulates the proliferation of early OCPs. **A** TRAP staining showed the TRAP( +) cells in trabecular area at D21 after injection of MC-38 in WT mice or IL4^KO^ mice and the quantification of OC area in bone surface (Scale bar = 100 μm) (n = 9). **B** TRAP staining showed the TRAP( +) cells in trabecular area at D21 after injection of CT-26 after treatment with vehicle or IL-4 neutralizing antibody and the quantification of OC area in bone surface (Scale bar = 100 μm) (n = 9). **C** Flow cytometry plots and quantification of the percentage of early OCPs at each timepoint after injection of MC-38 cells into tibias and the quantification (n = 3–9 per condition, each sample was pooled from 2 mice). **D** Flow cytometry plots and quantification of the percentage of BrdU( +) early OCPs at each timepoint after injection of MC-38 cells into tibias and the quantification (n = 3–6, each sample was pooled from 2 mice). **E** Flow cytometry plots and quantification of the percentage of BrdU( +) early OCPs in WT mice and IL4^KO^ mice at 10 days post injection of MC-38 cells. (n = 4, each sample was pooled from 2 mice). **F** Flow cytometry plots and quantification of the percentage of BrdU( +) early OCPs cultured in MC-38 CM after stimulated by vehicle or IL-4. (n = 3) **G** Flow cytometry plots and quantification of the percentage of BrdU( +) early OCPs cultured in MC-38 CM with or without IL-4 neutralizing antibody. (n = 6) **H** Flow cytometry plots of the percentage of IL-4 ( +) cells in neutrophils, macrophages or neutrophils, respectively. *p < 0.05, **p < 0.01

Next, we investigated how IL-4 impacted the bone destruction during bone metastasis of CRC. Since osteoclasts (OCs) were responsible for osteolysis in tumor metastasis and IL-4 was closely associated with OC formation, we compared the number of OCs between WT mice and IL4^KO^ mice after injection of MC-38 cells into tibias. Amazingly, TRAP staining showed the percentage of osteoclast surface in bone surface significantly reduced in IL4^KO^ mice comparing with WT mice at 3 weeks (Fig. 2A). Similarly, administration of IL-4 neutralizing antibody can also efficiently decrease the area of OCs comparing to that in control group at 3 weeks after injection of MC-38 cells (Fig. 2B). When there was blockage of IL-4 in bone metastasis of CT-26 cells, the area of OCs also decreased (Additional file 1: Fig S1A). These results illustrated that the effect of IL-4 on bone destruction may depend on regulating OCs.

Then, we detected whether IL-4 directly regulated the differentiation of OCPs. Unfortunately, treatment with recombinant IL-4 protein did not stimulate the osteoclast formation in the presence of RANKL and M-CSF as well as MC-38 CM (Additional file 1: Fig S1B), revealing that the effects of IL-4 on OCs should not depend on regulation of osteoclast differentiation. Since the number of OCs may also be determined by the number of OCPs, we detected the dynamic change of OCPs from D0 to D21 in bone metastatic model of MC-38 cells. Interestingly, we found CD115( +) OCPs reached the peak until 10 days post injection and then gradually decreased, which was similar with the changes of IL-4 (Fig. 2C), implying that IL-4 may participate in the increase of OCPs. Consistently, BrdU incorporation in OCPs also increased from D0 to D10 in both MC-38 induced bone metastasis (Fig. 2D). However, the percentage of apoptotic OCPs were slightly changed (Additional file 1: Fig S1C). These data implied that IL-4 may regulate the proliferation of OCPs. To verify our speculation, we detected the proliferative capacity of OCPs in IL-4^KO^ mice. As expected, BrdU incorporation in OCPs decreased nearly 50% in IL4^KO^ mice compared to that in WT mice at 10 days post injection of MC-38 (Fig. 2E). When using recombinant IL-4 stimulated OCPs cultured in MC-38 CM, BrdU incorporation obviously increased in IL-4 treated group (Fig. 2F). Then, we injected CT-26 cells intratibially to test the dynamics of OCPs. The results showed BrdU incorporation in OCPs significantly increased at D10 in contrast with that in normal mice (Additional file 1: Fig S1D). Bloackage of endogenous IL-4 could significantly prevented BrdU incorporation in OCPs in bone metastasis of CT-26 cells (Additional file 1: Fig S1E). These data indicated that IL-4 directly stimulated the proliferation of OCPs.

Then, we explored the source of endogenous IL-4. Firstly, we detected whether IL-4 derived from MC-38. The OCPs were cultured in MC-38 CM with or without IL-4 neutralizing antibody. Unexpectedly, no significant differences of BrdU incorporation can be observed between the IL-4 neutralizing antibody treated group and control group, implying IL-4 may not derive from MC-38 cells (Fig. 2G). Since immune cells were reported to be a dominant source of IL-4 (Balmer and Devaney 2002; Heredia et al. 2013), we detected the expression of IL-4 in macrophages, neutrophils and neutrophils in bone marrow through using flow cytometry, the results showed neutrophils labelled with CD45( +):CD11b( +):Ly6G( +), had the highest percentage of IL-4 expressing comparing with other cell types, indicating that IL-4 in bone marrow dominantly came from neutrophils (Fig. 2H and Additional file 1: Fig S2A).

### IL-4 regulates the proliferation of OCPs through IL4Rα

**Fig. 3:**
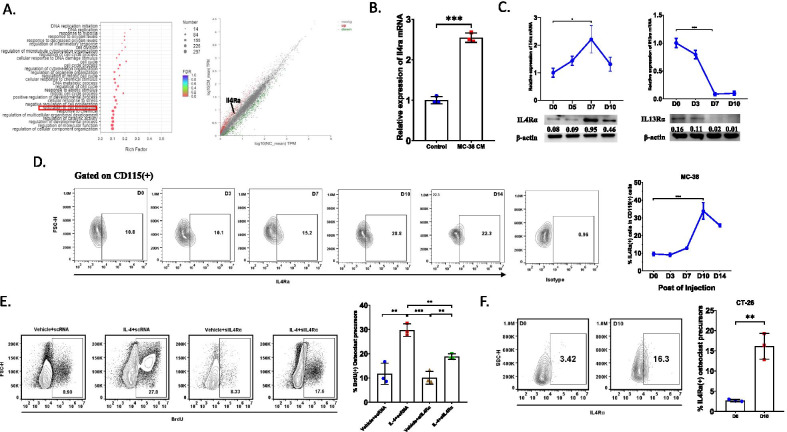
IL4Rα regulating the proliferation of early OCPs in metastatic bone of CRC. **A** Transcriptomic profiling revealing most changed pathways analyzed by KEGG pathway analysis (left) and scatter plot showing the differentially expressed genes (right). **B** qRT-PCR analysis detected the mRNA expression of IL4Rα in early OCPs cultured in MC-38 conditioned medium. (n = 3) **C** qRT-PCR analysis and western blots showing the mRNA level and protein level of IL4Rα or IL13rα in early OCPs isolated from bone marrow at each timepoint after injection of MC-38 cells. (n = 3–5 per condition, each sample was pooled from 3 mice) **D** Flow cytometry plots and quantification of the percentage of IL4Rα ( +) early OCPs isolated from bone marrow at each timepoint after injection of MC-38 cells. (n = 6–9 per condition) **E** Flow cytometry plots and quantification of percentage of BrdU( +) OCPs cultured in MC-38 CM after treated by recombinant IL-4 protein with/without transfection with IL4Rα-siRNA. (n = 3) **F** Flow cytometry plots and quantification of the percentage of IL4Rα ( +) early OCPs isolated from bone marrow at D0 and D10 after injection of CT-26 cells. (n = 3) *p < 0.05, **p < 0.01, ***p < 0.001

Then, we detected the downstream of IL-4 in regulating the proliferation of OCPs. Early OCPs were isolated from bone marrow at D0 and D10 after injection of MC-38 cells and the transcriptomic profiling was analyzed. KEGG pathway analysis showed genes enriched in “regulation of cell proliferation” pathway, and the expression of IL4Rα increased in OCPs at D10 (Fig. 3A). Since IL4/IL4Rα signal was reported to regulate cell proliferation (Heredia et al. 2013), we next analyzed the expression of IL4Rα in early OCPs during tumor microenvironment. The mRNA level of IL4Rα in OCPs evidently increased in CM-treated group compared to that in control group (Fig. 3B). In vivo, qRT-PCR analysis and Western blotting showed the mRNA and protein level of IL4Rα in OCPs increased gradually before 7 d.p.i and then decreased (Fig. 3C, left). As it was reported that there are two types of IL-4 receptor, Type I receptor consist of IL4Rα and γ-chain, Type II receptor contain IL4Rα and IL13Rα, thus we further explored the mRNA and protein expression of IL13Rα in early OCPs, unlikely IL4Rα, the mRNA and protein level of decreased significantly once CRC cells went into bone (Fig. 3C, right), indicating that Type 1 IL4 receptor dominantly participated in the bone metastasis of CRC.

Then, we detected the dynamic changes of the expression of IL4Rα protein in OCPs in vivo. Flow cytometry analysis revealed that the percentage of IL4Rα( +) early OPCs increased significantly during 10 days post injection of MC-38 and then decreased gradually (Fig. 3D). We noticed that this change was similar with the trajectory of the BrdU incorporation in early OCPs. To confirm whether IL-4 regulated the proliferation of early OCPs through IL4Rα, OCPs were transfected with IL4Rα siRNA to inhibit the expression of IL4Rα. Western blots showed transfection of IL4Rα siRNA efficiently downregulated the protein level of IL4Rα in OCPs (Additional file 1: Fig S2B). Then, OCPs were stimulated with IL-4 after transfection wit IL4Rα siRNA in culture of MC-38 CM. The results showed IL-4 promoted the BrdU incorporation in OCPs, which was consistent with previous findings. However, this effect was abolished after transfection with IL4Rα siRNA (Fig. 3E), indicating IL4Rα was indispensable for IL-4-induced proliferation of OCPs. Similarly, we also found the percentage of IL4Rα( +) OCPs was significantly higher at D10 compared to that in D0 in bone metastasis of CT-26 cells (Fig. 3F). These data together indicated that IL4Rα was a key regulator for IL-4 induced proliferation of OCPs in bone metastasis of CRC.

Then, we explored whether IL4Rα could be a potential therapeutical targets for treatment of bone metastasis of CRC. We injected of IL4R siRNA intratibially into CRC metastatic model of MC-38 cells or CT-26 cells. TRAP staining showed the number of osteoclasts decreased in IL4Rα siRNA treated group comparing with the number in control group at 21 DPI (Additional file 1: Fig S3A). Consistently, Safranin O staining revealed osteolysis was alleviated after treatment with IL4Rα siRNA (Additional file 1: Fig S3B). These results indicated downregulation of IL4Rα can significantly attenuated the osteoclast formation in vivo.

### ERK pathway mediates IL4/IL4Rα regulated proliferation of early OCPs

**Fig. 4:**
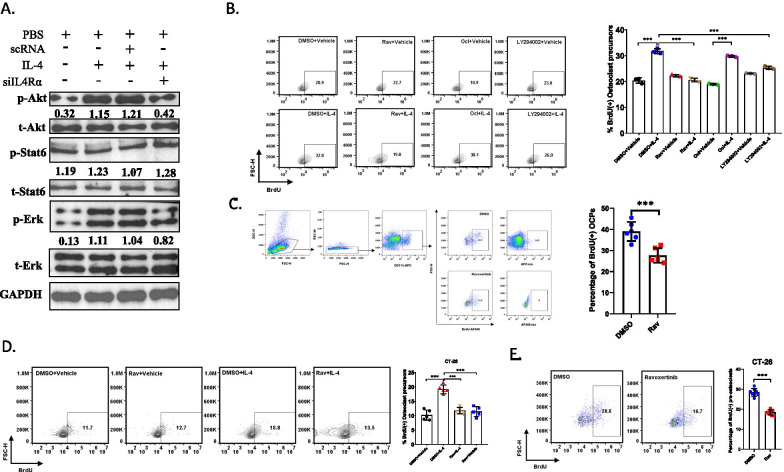
ERK pathway regulating IL4/IL4Rα-mediated proliferation of early OCPs. **A** Western blotting showed the protein level of ERK pathway, JAK-STAT pathway and PI3K-AKT pathway. (n = 3) **B** Flow cytometry plots and quantification of BrdU( +) early OCPs after treated by recombinant IL-4 protein with/without each antagonist of ERK pathway, JAK-STAT pathway and PI3K-AKT pathway, respectively. (n = 3) **C** Flow cytometry plots and quantification of the percentage of BrdU( +) early OCPs in bone marrow at 10 days post injection of MC-38 cells after treated by Ravoxertinib. (n = 6, each sample was pooled from 3 mice) **D** Flow cytometry plots and quantification of percentage of BrdU( +) early OCPs cultured in CT-26 CM after treated with IL-4 plus Ravoxertinib or not. (n = 5) **E** Flow cytometry plots and quantification of the percentage of BrdU( +) early OCPs in bone marrow at 10 days post injection of CT-26 cells after treated by Ravoxertinib. (n = 8, each sample was pooled from 3 mice) *p < 0.05, **p < 0.01, ***p < 0.001. *Rav* Ravoxertinib, *Ocl* Oclacitinib

Next, we explored the underlying mechanism regulating the proliferation of early OCPs mediated by IL4/IL4R signals. It was reported that ERK pathway, JAK/STAT pathway and PI3K/AKT pathway may participate in IL-4 mediated proliferation in various cell types (Heredia et al. 2013; Keijzer et al. 2011; Malabarba et al. 1995), thus we firstly tested which pathways were regulated by IL4/IL4R signaling in early OCPs. Western blots showed IL-4 activated the expression of AKT and ERK but not STAT6 and inhibiting IL4R expression can downregulate the protein level of phosphorylation of AKT and ERK (Fig. 4A), indicating overexpression of IL-4 stimulated ERK pathway and PI3K-AKT pathway but had less effects on activation of JAK-STAT pathway. Then we further investigated the role of these three pathways on the proliferation of early OCPs. We blocked these three pathways by using their antagonists, respectively, and tested the percentage of BrdU( +) early OCPs. Flow cytometry analysis indicated that BrdU( +) early OCPs decreased significantly when treated with Ravoxertinib, the ERK pathway antanogist, comparing with IL-4 treated group, While LY294002 just slightly downregulated the percentage of BrdU( +) early OCPs (Fig. 4B), indicating ERK pathway was the dominant regulator for IL4-mediated proliferation of early OCP s and AKT pathway partially participated in this process. To investigate whether blockage of ERK pathway could also prevent the proliferation of early OCPs in vivo, Ravoxertinib (10 mg/kg) was intratibially injected once per three days and the BrdU( +) early OCPs at 10 DPI were detected through flow cytometry, as expected, the proliferation of early OCPs was remarkedly prevented in Ravoxertinib-treated group comparing with control group (Fig. 4C). Similarly, when using Ravoxertinib treated OCPs cultured in CT-26 CM, the BrdU incorporation significantly upregulated in IL-4-treated group, but this trajectory could be efficiently blocked after stimulating with Ravoxertinib (Fig. 4D). When there was an intravital injection of Ravoxertinib in bone metastasis of CT-26 cells, the BrdU incorporation of OCPs could be prevented in Ravoxertinib-treated group at D10 (Fig. 4E). These data indicated Ravoxertinib could efficiently reverse the IL-4 mediated proliferation of OCPs both in vitro and in vivo.

### Blockage of ERK pathway significantly restored bone volume in bone metastasis of CRC

**Fig. 5:**
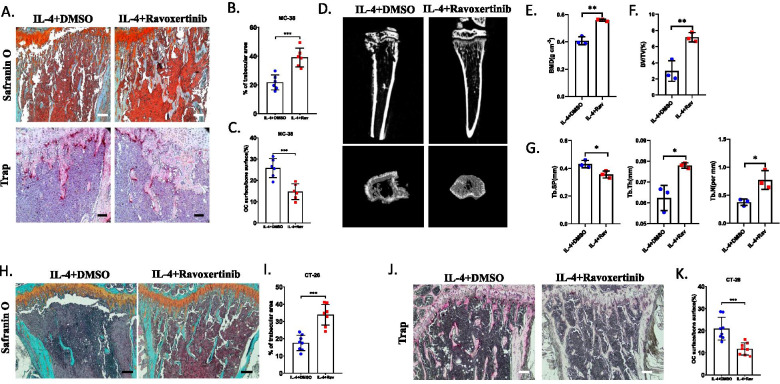
Blockage of ERK pathway significantly attenuating osteolysis in metastatic bone of CRC. **A**–**C** Histochemistry analysis and TRAP staining showed the bone resorption in tibias at 21 days post injection of MC-38 cells after administration of Ravoxertinib in the presence of IL-4 (Scale bar = 100 μm) (**A**) and the quantification of trabecular area (**B**) and the quantification of area of OCs (**C**). (n = 6) **D**–**E** μCT analysis showed the bone resorption in tibias at 21 days post injection of MC-38 cells after administration of Ravoxertinib in the presence of IL-4 (**D**) and compared the bone mineral density (BMD) of tibias at 21 days post injection of MC-38 cells after administration of Ravoxertinib in the presence of IL-4 (**E**). (n = 3) **F**, **G** Quantification of trabecular bone volume fraction (BV/TV) (**F**), trabecular thickness (Tb.Th), trabecular number (Tb. N) and trabecular separation (Tb. Sp) (**G**) of tibias at 21 days post injection of MC-38 cells after administration of Ravoxertinib in the presence of IL-4. (n = 3) (**H**–**I**) Histochemistry analysis showed the bone resorption in tibias at 21 DPI after injection of CT-26 cells in IL-4 treated mice with/without treatment of Ravoxertinib (Scale bar = 100 μm) (**H**) and quantification of trabecular area (**I**). (n = 8) (**J**–**K**) TRAP staining showed the area of OCs at 21 days post injection of CT-26 cells  (Scale bar = 100 μm) (**J**) and the quantification of OC area in bone surface  (**K**). (n = 8) *p < 0.05, **p < 0.01

To investigate whether blocking ERK pathway had the function to attenuate IL-4 mediated bone resorption, Ravoxertinib was injected with IL-4 intratibially. At 3 weeks post injection, histochemistry analysis and μCT showed Ravoxertinib significantly restored the bone volume and prevent the activation of osteoclasts, bone mineral density and the volume of trabecular increased after treatment of Ravoxertinib (Fig. 5A–G). Consistently, Ravoxertinib treatment could also prevent bone resorption and osteoclast activation in bone metastasis of CT-26 cells (Fig. 5H–K). These results showed blockage of EKR pathway efficiently attenuated IL-4 induced osteolysis in bone metastasis of CRC.

## Discussion

In osteolytic cancer, abnormal activation of osteoclasts is critical to the bone metastasis and tumor progression (Grano et al. 2000; Hayes et al. 2018). However, it is still unclear how the osteoclasts or the precursors are regulated by cancer cells in early stage of bone metastasis. On the other hand, colorectal cancer is considered as one of classical cancers that promoting bone resorption, but little is known about the microenvironment changes after bone metastasis of CRC. In this study, we have explored that CRC cells can promote the proliferation of early OCPs once they are transplanted into bone. The viability of early OCPs has enhanced before 10 DPI, since we previously demonstrated that obvious osteolysis could be observed at about 2 weeks post injection, and thus the proliferation of early OCPs occurred at early stage of bone metastasis of CRC. In addition, comparing with proliferative capacity, the apoptosis of early OCPs was limited changed during this process, and thus it indicated that CRC cells dominantly promoted the number increasing of early OCPs through regulating proliferation. Our study firstly revealed microenvironment changes after bone metastasis of CRC at early stage.

IL-4 was reported to prevent the osteoclastogenesis in numerous studies (Mangashetti et al. 2005), while Dou et al. explored that IL-4 induced M2 macrophages tended to be more likely differentiate into osteoclasts comparing with M1 macrophages in osteoporosis model (Dou et al. 2018). In this study, we found IL-4 had few effects on osteoclastogenesis in the presence of RANKL in vitro experiments, whereas the number of osteoclasts was significantly influenced in the presence of IL-4 during bone metastasis of CRC in vivo, and thus we thought IL-4 enhanced the number of osteoclasts indirectly by promoting the proliferation of early OCPs. The increased number of early OCPs stimulated by IL-4 provided plenty of “seeds” for osteoclastogenesis in later stage. In fact, IL4/IL4R signal positively or negatively regulated the proliferation of several cell types. IL-4/IL-13 increased the proliferation of human colon cancer cells dependent on NAPDH oxidase 1, endometriotic stomal cells, mast cells, human lymphoblasts, fibroblasts, fibro/adipogenic progenitors (FAPs) (Heredia et al. 2013; Liu et al. 2017; Monroe et al. 1988; Chaikin et al. 1990; OuYang et al. 2008). On the other hand, the proliferation of breast cancer cells, retinal progenitor cells, human astrocytes, preadipocytes could be inhibited by IL-4, and it remained controversy on its effects on proliferation of B cells (Silva et al. 2008; Estes et al. 1993; Hua et al. 2004; Blais et al. 1996; Llorente et al. 1990). These studies showed a dual effect of IL-4 on cancer cells and other cell types. In this study, we demonstrated that IL-4 could stimulate the proliferation of early OCPs in CRC condition through a IL4R-dependent way. Although IL-13 was also reported to participate in IL-4 mediated cell proliferation (Heredia et al. 2013), we here found Type 1 IL-4 receptor but not IL13 receptor was responsible for proliferation of early OCPs. PI3K-AKT pathway, JAK-STAT pathway and MAPK pathway can potentially be the downstream of IL4/IL4R signal (Heredia et al. 2013; Keijzer et al. 2011; Malabarba et al. 1995; Friedrich and Wietek 2001), we revealed that the blockage of ERK pathway most significantly inhibited the IL-4-mediated proliferation of early OCPs, while AKT pathway also partially regulated the proliferation, indicating IL-4 regulated the proliferation of early OCPs during bone metastasis of CRC through several ways and ERK pathway could be the most important regulator. Importantly, blockage of ERK pathway by using its antagonist, Ravoxertinib, could efficiently counter IL-4-induced bone osteolysis, showing the potential therapeutical efficiency of Ravoxertinib on treatment of osteolysis induced by IL-4.

Several immune cell types were explored to be the source of IL-4, including neutrophils, NK T cells, masts (Balmer and Devaney 2002; Heredia et al. 2013; Horsmanheimo et al. 1994). The study here showed neutrophils could be the dominant source of IL-4 in bone metastasis of CRC, and flow cytometry analysis found the percentage of neutrophils was higher than the percentage of neutrophils or macrophages, as well as the neutrophils positive for IL-4 which was also higher than the expression of IL-4 in neutrophils or macrophages. Neutrophils were found to regulate the pre-metastatic niche formation and to promote the bone metastatic progression of prostate cancer (Costanzo-Garvey et al. 2020; Jablonska et al. 2017), which was consistent with previous findings. Our results supported and firstly identified that neutrophils were critical to the bone metastatic niche formation caused by CRC. In addition, bone metastasis of CRC often occurred in elder people, the immune microenvironment may differ from that in younger people. In this study, younger mice were used in our research, like some other studies regard to bone metastasis research (Jiang et al. 2019; Ren et al. 2019). Considering aging as a potential confounder for tumor microenvironment, elder mice should be used to verify the effect of IL-4 on bone metastasis of CRC in further study.

## Conclusion

**Fig. 6:**
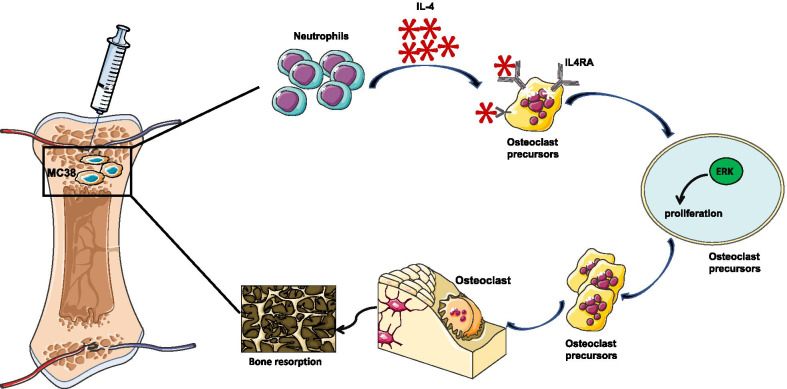
Schematic of IL-4 regulating the proliferation of OCPs and facilitating osteolysis in bone metastasis of CRC

To our best knowledge, this is the first study to explore the effect of IL-4 on proliferation of early OCPs in bone metastasis of CRC, and we have revealed the indispensable role of neutrophil-derived IL-4 on metastatic niche formation in early stage of bone metastasis of CRC. Furthermore, the ERK pathway is the dominant downstream of IL4/IL4R signal during this process, targeting the activation of ERK pathway can efficiently prevent bone resorption of CRC (Fig. 6). Our study has detailly explored the biological behavior of OCPs at early stage of bone metastasis of CRC and has provided a new potential therapeutical target for restoring bone volume in osteolytic cancer.

## Supplementary Information

**Additional file1: Fig. S1.** IL-4 promotes the proliferation of OCPs in bone metastasis of CRC. (A) TRAP staining showed the OC activation in bone metastasis of CT-26 cells after treated with IL-4 nAb or vehicle at D21. (n = 9) (B) TRAP staining showed the OC formation treated with IL-4 or vehicle and quantification of number of OCs. (n = 6) (C) Flow cytometry plots and quantification of apoptotic CD115( +) early OCPs at each timepoint after injection of MC-38 cells through using TUNEL assay. (n = 3–4 per condition) (D) Flow cytometry plots and quantification of BrdU( +)early OCPs at D0 and D10 after injection of CT-26 cells into tibias. (n = 6) (E) Flow cytometry plots and quantification of BrdU( +) OCPs in tibias at D10 treatment with IL-4 nAb after injection of CT-26 cells. (n = 6) ***p < 0.001. **Fig. S2.** Gating strategy of myeloid cells in bone marrow. (A) Flow cytometry plots of myeloid cells in bone marrow. (B) Western blots showed the protein level of IL4Rα in OCPs after transfection with siILR4α or scRNA. (n = 3). **Fig. S3.** Targeting IL4Rα attenuates the osteolysis in bone metastasis of CRC. (A) TRAP analysis showed OCs in bone surface at 21 days post injection of MC-38 cells or CT-26 cells in siIL4Rα treated group compared with that of control group and the quantification of the area of OCPs in bone surface (Scale bar = 100 μm). (n = 6) (B) Safranin O and fast green staining revealed the trabecular area at 21 days post injection of MC-38 cells or CT-26 cells in siIL4Rα treated group compared with that of control group and the quantification of the trabecular area (Scale bar = 100 μm). (n = 6) **p < 0.01, ***p < 0.001.

## Data Availability

All data generated or analyzed during this study are included in this published article and its additional information files.

## Abbreviations

CRC: Colorectal cancer
OCP: Osteoclast precursors
M-CSF: Colony stimulating factor
CM: Conditioned medium
DMEM: Dulbecco's modified Eagle's medium
TRAP: Tartrate resistant acid phosphatase
PVDF: Polyvinylidene difluoride
BSA: Albumin bovine
TBS: Tris buffered saline
ELISA: Enzyme-linked immuno sorbent assay
